# A powerful association for comorbidity analysis based on score based test

**DOI:** 10.1186/1471-2105-14-S17-A4

**Published:** 2013-10-22

**Authors:** Zhifa Liu

**Affiliations:** 1 Department of Biostatistics, St Jude Children’s Research Hospital, Memphis, TN 38105, USA

## Background

In the studies of mental and behavioral disorders, comorbidity is an important issue since multiple correlated disorders are usually recorded to understand the etiology of substance dependence, which is imperative to the development of effective treatment and prevention strategies. Many studies have been reported the comorbidity between substance abuse disorders and psychiatric disorders such as anxiety and major depression. In order to investigate the association between the comorbidity of complex diseases and genetic locus, it is critical to develop a computationally efficient and powerful multiple traits association test. Recently, taking the advantage of high throughput genomic data, lots of genetic variants have been identified for individual drug addiction based on genome-wide association studies (GWAS). Despite many successes, the current GWAS may be insufficient to detect genetic variants with moderate-to-small effect since a stringent significance threshold is used to control the false discovery rate, which is a key factor to the missing heritability problem.

## Materials and methods

To detect novel genetic variants, joint analyzing correlated traits are promising solutions. First, joint analyzing correlated traits may increase the power in detecting genetic variants with moderate effects across multiple traits by exploiting the correlation between traits. Second, joint analysis can alleviate multiple comparison problems which incurred in analyzing individual trait separately. Comprehensive studies have been used to demonstrate that jointly testing correlated traits is more powerful than testing a single trait at a time. Therefore, it is important to consider the comorbidity of correlated traits in order to detect novel genetic loci in GWAS. We introduce a multiple traits association test based on least square. The proposed method only needs to specify the marginal distribution of multiple traits. We systematically investigate the strengths and weaknesses of the multiple traits association test through simulation and comprehensive real data analysis. We demonstrated the advantage of multiple traits association test when multiple traits share common genetic variation. However, when multiple traits share no or weak common genetic variation, the multiple traits association test has no advantage.

